# Are dogs red–green colour blind?

**DOI:** 10.1098/rsos.170869

**Published:** 2017-11-08

**Authors:** Marcello Siniscalchi, Serenella d'Ingeo, Serena Fornelli, Angelo Quaranta

**Affiliations:** Department of Veterinary Medicine, Section of Behavioral Sciences and Animal Bioethics, University of Bari ‘Aldo Moro’, Bari, Italy

**Keywords:** dog, colour vision, physiology, behavioural method

## Abstract

Neurobiological and molecular studies suggest a dichromatic colour vision in canine species, which appears to be similar to that of human red–green colour blindness. Here, we show that dogs exhibit a behavioural response similar to that of red–green blind human subjects when tested with a modified version of a test commonly used for the diagnosis of human deuteranopia (i.e. the Ishihara's test). Besides contributing to increasing the knowledge about the perceptual ability of dogs, the present work describes for the first time, to our knowledge, a method that can be used to assess colour vision in the animal kingdom.

## 1. Introduction

Dogs' retinal structure clearly provides the potential for colour vision [1,2]. Specifically, visual-evoked potential [3,4] and immunohistochemical [1] studies have demonstrated that dogs possess two classes of cone pigments, one sensitive to long/medium wavelength light (555 nm spectral sensitivity; red/green) and the other sensitive to short wavelength light (429 nm spectral sensitivity; blue). The presence of these two discrete cone subtypes indicates a potential dichromatic vision. Concerning visual acuity, dogs are less able than humans to perceive clearly all the details of an object (four to eight time worse than humans) [5,6]. This is owing to the different neural structures of the dogs' eyes and in particular to the fewer connections of the rods to the ganglion cells and the smaller number of optic nerve fibres [5]. Furthermore, dogs can discriminate brightness differences but their ability is about two times worse than in humans [7].

Although early behavioural studies on dogs' colour vision produced conflicting results (reviewed by [8]), recent behavioural studies support the presence of dichromatic vision in canine species, indicating that colour cues are important for dogs during their normal activities under natural photonic lighting conditions [9].

Colour vision tests in the animal kingdom include both spontaneous and learned behaviour [10]. However, the most employed technique of testing colour vision in dogs uses associative learning with a food reward [9,10,11]. Using this procedure, Kasparson *et al*. [9] recently showed that colour proved to be more informative than brightness when dogs choose between visual stimuli differing both in brightness and hue. Associative learning was also used by Neitz *et al*. [11] to study different wavelength colour matching in three adult pure breed dogs. Overall, results of this work supported the hypothesis that colour perception is essential for canine vision and that it is dichromatic in character. In addition, computer estimation of the spectral sensitivity of the two photopigments of the dog's retina suggested that dichromatic vision in canine species resembles that of human deuteranopia (i.e. red–green colour blindness).

In order to directly test this hypothesis, we used to our knowledge, for the first time, an orienting response (e.g. movements of the eyes, the head and the whole body) to movements of a coloured target in the dog's visual field. The employment of unlearned response has a clear advantage because no preliminary training is required prior to the colour vision test, allowing the testing of a large number of subjects in a short period of time and avoiding motivational and reinforcement issues typical of learned response (e.g. food reward occurring during associative learning).

Investigating the understanding of colour perception/blindness in dogs is particularly interesting for two main reasons: (i) the dog is an important animal model of human retinal genetic disorders [1]; and (ii) the dog plays a number of significant roles within the human community (e.g. animal assisted therapy, search and rescue work and as guide dogs for visually-impaired humans) often requiring the use of visual cues.

Furthermore, considering that dogs' vision is weaker than the human one, this could affect their responses in an ethological experiment [6], deepening the understanding of colour perception could be decisive in the design of visual tasks suitable for dogs' visual capabilities.

## 2. Material and methods

### 2.1. Subjects

Subjects were 21 domestic dogs of various breeds. We excluded six dogs: four dogs, because after hearing the beep used to capture their attention on the screen, they did not look at any stimuli; two dogs were potentially influenced by the owner during the test (i.e. the owner repositioned the dog's head to the screen). Hence the final sample consisted of 16 dogs (three Australian shepherds, one Épagneul Breton, one Weimaraner, one Labrador retriever and 10 mixed-breed dogs). Dogs ranged from 1 to 8 years of age (2.00 ± 1.96; mean years ± s.d). All dogs (nine females and seven males) were pets living in households. Only one male and five females were desexed. No subject had been tested previously.

### 2.2. Experimental setup

The experiment was carried out at the Department of Veterinary Medicine of Bari University, in Italy, in a rectangular room (5.85 m long, 3.50 m wide) isolated from the rest of the Department in order to avoid any noise interference.

Visual stimuli were presented on a large screen display homogeneously illuminated (Nec Multisync V321^®^ 32^″^ with a refresh rate of 85 Hz and a resolution of 1280 × 1024 dpi), which was placed on one side of the testing room. Apart from the light arising from the monitor screens (163 lux measured approximately by the distance of the dog's head from the screen), the room was maintained under natural light conditions. The experiment was carried out throughout the daylight hours. The light penetrated into the room through two vertical windows located on the two sides of the screen and 1 m behind it. During the test, the average brightness of the room was 197 lux (max = 201/min = 195 lux) and no extra artificial lights were turned on. Dogs were led in the testing room 5 min before the beginning of the test in order to let them become accustomed to the light conditions of the room. Meanwhile, owners were informed about the aim of the study, the procedures and the order of the stimuli presentation. They were also asked to not interact with their dogs during the test and to stare at a fixed spot centrally located to the screen and about 20 cm above it, to avoid the fact that any involuntary cues provided by them could influence dogs' reactions to the stimuli.

In the testing room a chair for the dog's owner was placed at one side of the room, facing the screen at a distance of about 2 m and centrally positioned. The owner was asked to sit on the chair during the trials. The dog sat or was laid between the owner's legs, facing towards the screen on which animations were presented. Two digital video cameras were used to record the dogs' behavioural responses (Sony handycam HDR-XR550E, 25 fps). Synchronization of video cameras was carried out by starting each recording simultaneously with the use of a single remote control. The first camera was positioned behind the dog–owner dyad, facing the screen while the second camera was positioned in front of the dog above the screen in order to record subjects' spontaneous looking behaviour. Only the videos recorded by the latter were used for the data analysis. Since the end of the ‘beep’ sound signalled the stimulus appearance, the analysis was carried out considering the audio track of the video.

### 2.3. Choosing a valid target

**Figure 1.**
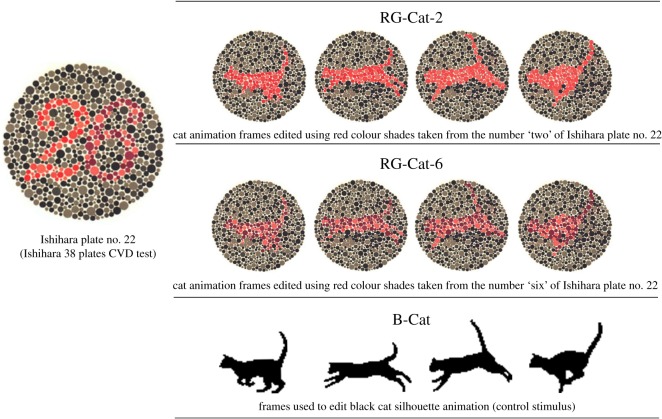
Ishihara plate no. 22 (Ishihara 38 plates for colour vision deficiency (CVD) test) and single frames used to edit, respectively, RG-Cat-2, RG-Cat-6 and B-Cat animations.

One of the goals of the orienting paradigm is that the behavioural response (e.g. movements of the eyes, the head and the whole body) of the subjects must be easily and clearly detectable. In order to verify these conditions, six dogs (one Irish setter, five mixed-breed dogs) aged between 2 and 13 years (6.33 ± 1.72; mean years ± s.e.m.) were preliminarily tested with two different black targets against a white background: (i) a black animated silhouette of the ‘running cat’ (moving target) and (ii) a black square of the same cat target's surface (fixed target). The ‘running cat’ (B-Cat) was obtained from the web. Four frames were required to cover the cat's entire running sequence (figure 1, B-Cat), then the digitalized sequence was looped and projected onto a computer screen.

Stimuli were displayed during the experiments as PowerPoint slideshows. The black silhouettes were presented on a white background. Both for the cat and the square an animated entrance from one side of the screen to the other with a linear velocity of 1.192 pixels s^−1^ was set. Side (left/right) and order (cat–cat–square–square or square–square–cat–cat) of entry was alternated over trials. Each stimulus was presented twice × each dog, for a total of four stimuli in each trial: two cats and two squares. The first, the last and in between stimuli slides were homogeneous black. The change between the black and stimulus slides (stimuli presentation) was controlled by the experimenter through a closed-circuit video system located in an adjacent room and was dependent on the attention of the dog to the screen. A ‘beep’ sound lasting 1 s was used to focus dogs' attention on the screen. Stimuli animations were displayed immediately after the end of the ‘beep’ sound. A loudspeaker placed centrally behind the monitor played the sound. The loudspeaker and the dog's head were all in a straight line in order to avoid any possible left–right dog's head orienting response owing to the beep sound and not to the visual stimuli. The change between the stimulus and the black slides (disappearance of the stimulus) was automatic after 3 s.

Audio–visual stimuli presentation was controlled by the experimenter through a computer from an adjacent room via a closed-circuit video system.

Data analysis revealed that although there was no difference in the reaction time (i.e. the time between the appearance of the target on the screen and the looking behaviour of the dog) between targets (B-Cat (2.15 ± 0.03; *m* ± s.e.m.) versus black square (2.05 ± 0.03; *m* ± s.e.m.) (*Z* = 16, *p* > 0.25; related samples Wilcoxon signed-rank test)), the score for alerting behavioural response was higher for the cat target (B-Cat (3.41 ± 0.59; *m* ± s.e.m.) with respect to the black square (1.25 ± 0.25; *m* ± s.e.m.) (Z = 21, *p* < 0.05; related samples Wilcoxon signed-rank test)). For this reason a ‘running cat’ animated silhouette was then used as the target stimulus.

### 2.4. Red–green blindness test

Since recent studies showed a same efficacy of printed and computer versions of the Ishihara plates in screening for human colour deficiency [12], different shades of colours were taken from computer version plates of Ishihara's tests for colour deficiency (figure 1). In particular, given that from the use of Ishihara's diagnostic test for human deuteranopia it appears that colour-blind subjects have difficulty in interpreting correctly the number ‘6’ depicted on Ishihara's plate no. 22 (i.e. people with normal vision read the number ‘26’ while red–green colour-blind subjects read only the number ‘2’), this plate has been used and modified for testing deuteranopia in dogs.

Two new animated cat targets were edited using eight frames (4 × each animation) in which red and green shades of Ishihara plate no. 22 were used.

In particular, the red–green cat ‘2’ (RG-Cat-2) and the red–green cat ‘6’ (RG-Cat-6) animations were edited using, respectively, the red shades employed to depict the number ‘2’ and the number ‘6’ of the Ishihara plate no. 22, both having the green shades of the same plate on background (figure 1: RG-Cat-2, RG-Cat-6).

Cats' silhouettes were then adapted frame by frame to Ishihara plates and digitalized/looped as previously reported for the ‘B-Cat’ target in order to create two new cat moving targets (see the electronic supplementary material videos RG-Cat-2 and RG-Cat-6).

We predicted that if dogs are red–green colour-blind most of the tested subjects should see and correctly interpret (showing orientation movements of the head and tip of predatory behaviour) the movements of the cat obtained by using the same colour shades employed to depict the number ‘2’ on the Ishihara's plate no. 22. On the other hand, dogs should show difficulties in interpreting the movements of the cat edited by using the same colour shades of the number ‘6’ taken from the same plate.

In addition, two different random animations were made using only the two shades of colours taken from the background of the Ishihara's plate no. 22 (green background plates: G-Background-1 and G-Background-2) in order to test whether the orienting dog's response was owing to the plate animation per se instead of to the perceived cat's movements (see the electronic supplementary material videos G-Background-1 and G-Background-2).

Five stimuli were displayed during each trial by PowerPoint slideshows on a white background and were presented in pairs: the first slide showed two G-Background-1 (control animated plates), the second and the third presentation showed, respectively, the animations of RG-Cat-6 versus G-Background-2 and RG-Cat-2 versus G-Background-2; finally the B-Cat animation was presented alone as a control.

Animations of RG-Cat-2 versus RG-Cat-6 were presented to dogs during a separate trial: the first slide showed two G-Background-1 (control animated plates), the second presentation showed, respectively, the animations of RG-Cat-2 versus RG-Cat-6; finally the B-Cat animation was presented alone as a control.

The change between stimuli (stimuli presentation) was controlled by the experimenter and was dependent on the attention of the dog to the screen (inter-stimulus presentation time: 7–120 s). The stimulus presentation procedure was the same as described in §2.3. The side of appearance of the ‘moving cat’ stimuli was randomized within each session. Owners were asked not to influence their dogs' behaviour (e.g. either to indicate the screen or to force looking behaviour). If the dog was distracted during the presentation of the stimuli or if it left the starting position (despite the beep sound) it was repositioned and the stimulus was then represented. Dogs visual angle was approximately of 2° 51′ 0.85^″^ and it was calculated using the following formula: visual angle = 2 × atan [(object size/2)/object distance].

The behaviour of the dogs was video recorded continuously during stimulus. Two trained observers who were blind to the testing paradigm subsequently analysed the video footage. The video was used to score any of the following listed behaviour: ears up-forward, scanning (dog turning head from left to right), eyes wide open, forward body orientation, eye/ear directed towards the target, gaze, head slightly lowered, paw lifted, freezing, alert position and head tilt. Each performed behaviour was allocated a score of 1, and the total for each dog was used to generate a reactivity index for the ‘alerting–targeting’ behavioural category.

**Figure 2.**
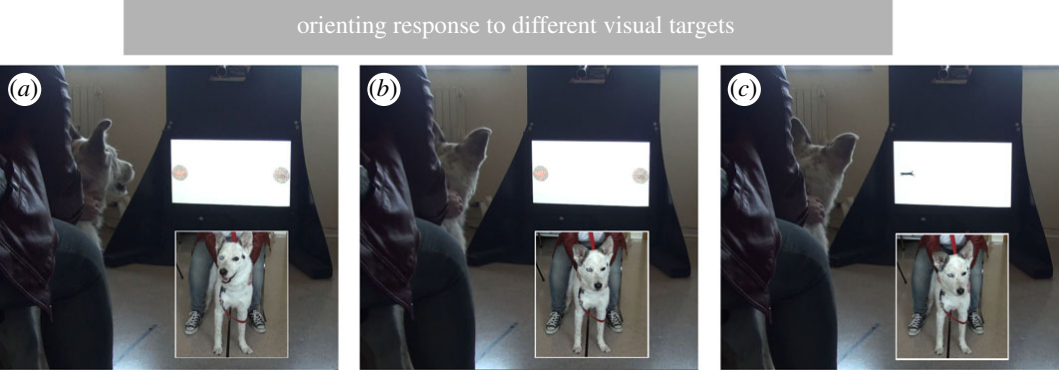
Experimental setup and orienting response to different visual targets: (*a*) RG-Cat-6; (*b*) RG-Cat-2; (*c*) B-Cat.

In addition, looking time to different visual stimuli was measured, analysing dogs behaviour from the beginning of the target animation (soon after the ‘beep’ sound) until the stimuli disappeared (figure 2 and electronic supplementary material video test).

Inter-observer reliability was assessed by means of independent parallel coding of a random sample of videotaped sessions (i.e. 70%) and calculated as percentage agreement (which was always higher than 94%).

A Sencore ColorPro 5 colorimeter sensor and Sencore ColorPro 6000 software were used to calibrate the colours of the monitor to CIE Standard Illuminant D65 (the mean, maximum and minimum CIEDE2000 monitor's values after colour calibration were respectively 0.67, 1.64, 0.35). The same apparatus was also used to measure the brightness of the white background and the average brightness of the tested stimuli (table 1).

**Table 1.** Brightness of tested stimuli: brightness of the white background and the average brightness of the tested stimuli (cd m^−2^).

brightness (cd m^−2^)	white background	G-Background-1	G-Background-2	RG-Cat-2	RG-Cat-6	A-Background-1	A-Background-2	A-Cat-2	A-Cat-6	B-Cat
mean	97.83	32.44	30.63	41.90	37.92	32.15	30.27	41.99	37.81	0.28
minimum	94.88	26.05	24.9	31.03	17.74	28.69	22.39	36.84	32.24	0.18
maximum	100.44	37.18	40.1	47.04	48.71	40.15	38.22	48.98	50.38	0.40

Furthermore, before testing, a computer version of Ishihara plate no. 22, RG-Cat-2 and RG-Cat-6 animations were directly presented to four human males clinically diagnosed with deuteranopia. All of the four red–green colour-blind subjects read only the number ‘2’ during inspection of the Ishihara plate no. 22 and recognized the moving cat during presentation of the RG-Cat-2 and not during RG-Cat-6.

### 2.5. Achromatic test

A subsample of nine dogs (five females and four males) was tested with achromatic versions of RG-Cat stimuli in order to see if subjects use achromatic cues to perceive cat's moving animations.

**Figure 3.**
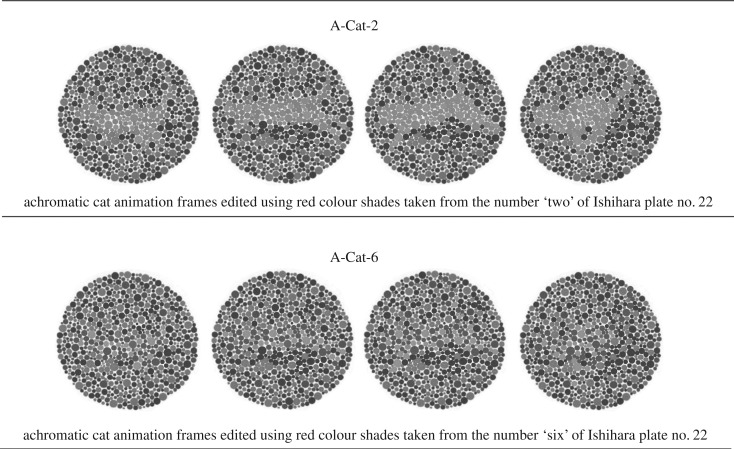
Achromatic stimuli: single frames used to edit respectively A-Cat-2 and A-Cat-6 animations.

Two new animated cat targets were edited using eight frames (4 × each animation) in which achromatic versions of red and green shades of Ishihara plate no. 22 were used (figure 3 and table 1).

In particular, the A-Cat-2 and the A-Cat-6 stimuli were edited using respectively the achromatic versions of the RG-Cat-2 and the RG-Cat-6 animations used during the red–green colour-blind test.

We predicted that if dogs use achromatic cues to perceive the cat animation most of the tested subjects should see and correctly interpret (showing orientation movements of the head and tip of predatory behaviour) more the movements of the cat obtained by using the achromatic version of the same colour shades employed to depict the number ‘2’ on the Ishihara's plate no. 22 with respect to the movements of the cat edited by using the achromatic version of the same colour shades of the number ‘6’ taken from the same plate (this prediction is based on the fact that achromatic contrasts are more apparent in the first condition than in the second).

In addition, two different random achromatic animations (i.e. A-Background-1 and A-Background-2) were made using G-Background-1, and G-Background-2 stimuli without colour cues.

Five stimuli were displayed during each trial by PowerPoint slideshows on a white background and were presented in pairs: the first slide showed two A-Background-1 (control animated plates), the second and the third presentation showed, respectively, the animations of A-Cat-6 versus A-Background-2 and A-Cat-2 versus A-Background-2; finally, the B-Cat animation was presented alone as a control on the same side of the A-Cat targets.

The testing procedure was identical to that described above for the red–green colour blind test.

The red–green colour blind and the achromatic tests were presented separately (at about a 1-week interval) and the presentation order was alternated between subjects (i.e. five subjects performed the red–green blindness test first and four dogs performed the achromatic test first).

## 3. Results

### 3.1. Red–green colour-blind test

**Figure 4.**
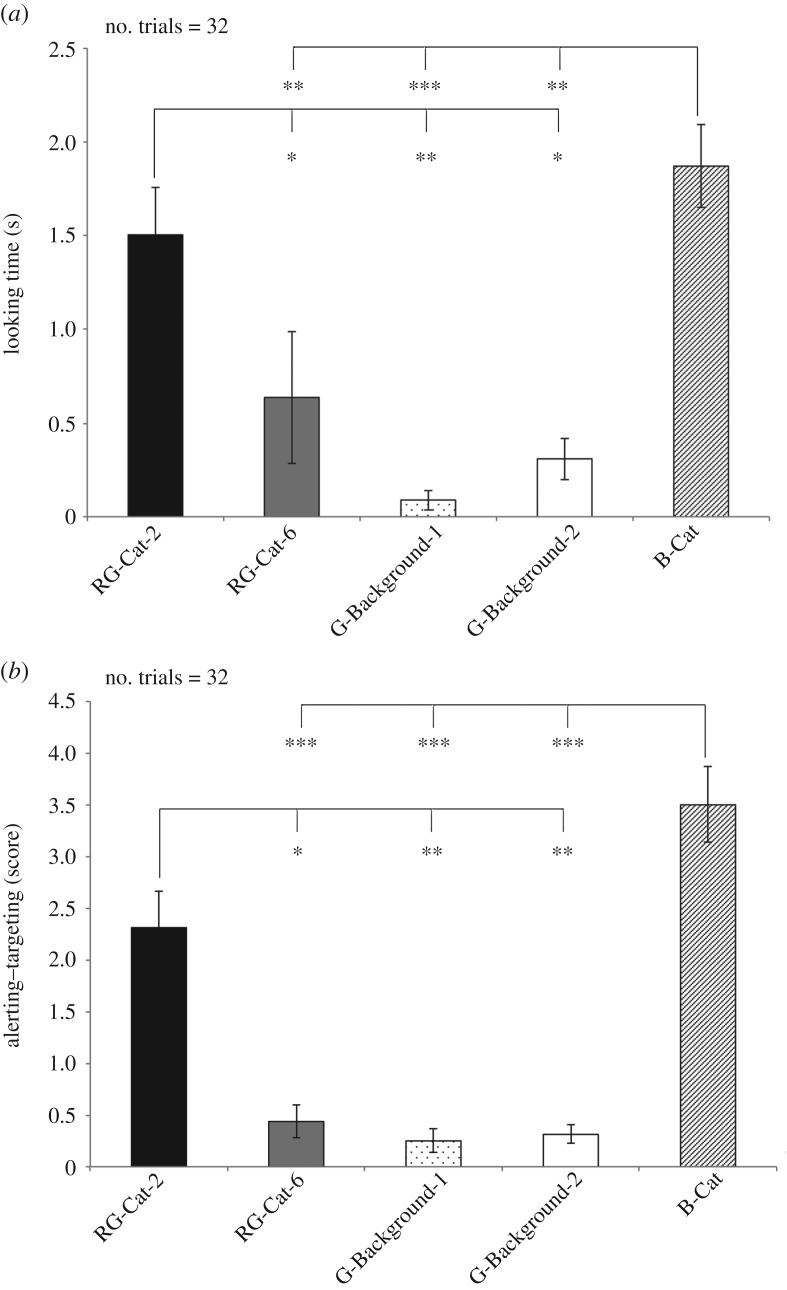
Red–green colour-blind test: looking time (*a*) and score for alerting–targeting behaviour (*b*) during presentation of different coloured visual stimuli (means with s.e.m. are shown; *p < 0.05, **p < 0.01, ***p < 0.001; Dunn's post hoc test).

Results for the red–green colour-blind test are shown in figure 4*a,b*.

Friedman's ANOVA revealed a main effect of the type of visual stimulus on the mean looking time ($\chi_{4}^{2}=35.986$, *p* < 0.001) (figure 4*a*). Dunn's post hoc test revealed that this main effect was owing to the time spent at looking both RG-Cat-2 and B-Cat being longer with respect to other stimuli: RG-Cat-2 (1.50 ± 0.25; *m* ± s.e.m.) versus G-Background-1 (0.09 ± 0.05 (s); *m* ± s.e.m.) (*p* < 0.01); RG-Cat-2 versus G-Background-2 (0.31 ± 0.11 (s); *m* ± s.e.m.) and versus RG-Cat-6 (0.63 ± 0.35 (s); *m* ± s.e.m.) (*p* < 0.05); B-Cat (1.87 ± 0.22 (s); *m* ± s.e.m.) versus G-Background-1 (*p* < 0.001); B-Cat versus G-Background-2 and RG-Cat-6 (*p* < 0.01). No statistical significant differences were found between the other pairwise comparisons (*p* > 0.05, Dunn's post hoc test).

As to the behavioural score, the analysis revealed that there was a statistical significant difference in the score for alerting behavioural response between visual stimuli ($\chi_{4}^{2}=45.356$, *p* < 0.001; Friedman's ANOVA, figure 4*b*). Dunn's post hoc test revealed that the RG-Cat-2 (2.31 ± 0.35; *m* ± s.e.m.) elicited a higher alerting behavioural response with respect to the G-Background-1 (0.25 ± 0.11; *m* ± s.e.m.), G-Background-2 (0.31 ± 0.08; *m* ± s.e.m.) (*p* < 0.01) and RG-Cat-6 (0.43 ± 0.15; *m* ± s.e.m.) (*p* < 0.05). Similarly, the analysis revealed higher alerting behavioural response between B-Cat and the other visual stimuli with the exception of RG-Cat-2: B-Cat (3.50 ± 0.36; m ± s.e.m.) versus G-Background-1, G-Background-2 and RG-Cat-6 (*p* < 0.001). No other significant statistical differences were found (*p* > 0.05 for all pairwise comparisons, Dunn's post hoc test).

### 3.2. Achromatic test

**Figure 5.**
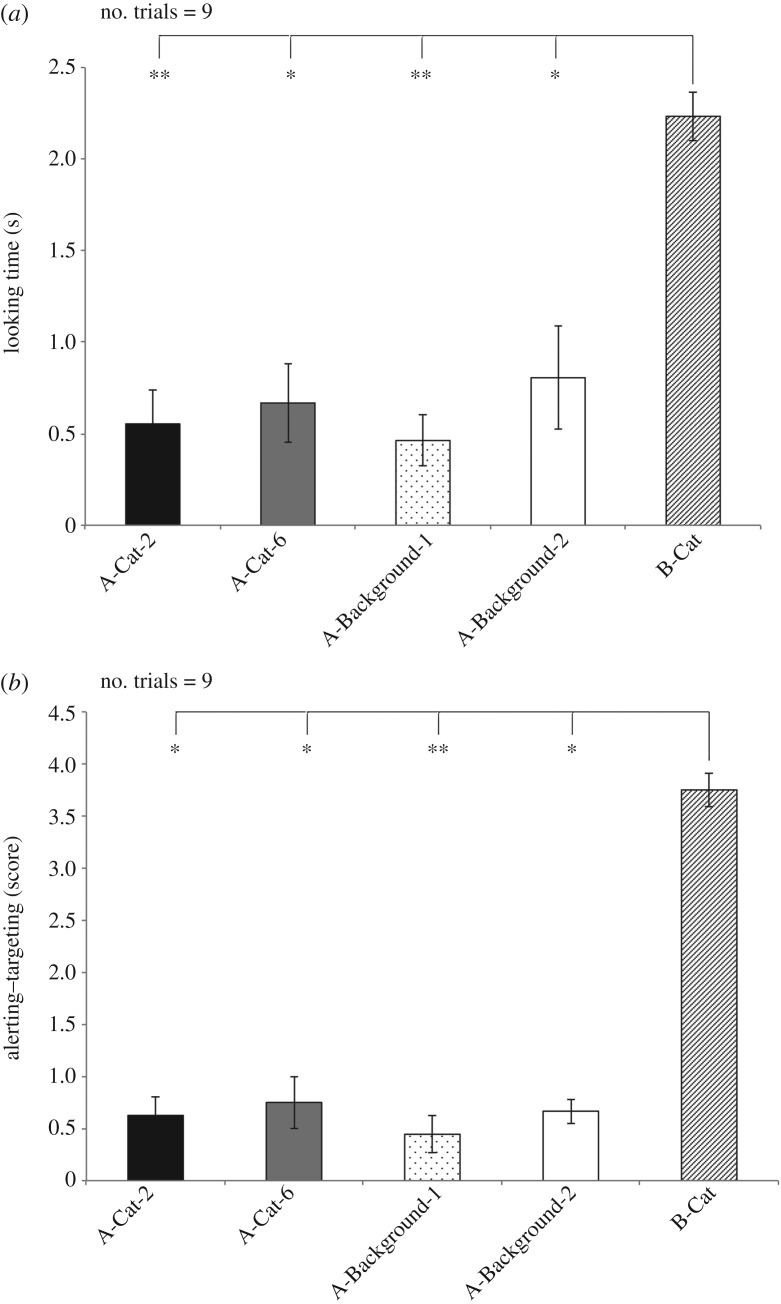
Achromatic test: looking time (*a*) and score for alerting–targeting behaviour (*b*) during presentation of different achromatic visual stimuli (means with s.e.m. are shown; *p < 0.05, **p < 0.01, ***p < 0.001; Dunn's post hoc test).

Results for the achromatic test are shown in figure 5*a*,*b*.

Friedman's ANOVA revealed a main effect of the type of visual stimulus on the mean looking time ($\chi_{4}^{2}=19.885$ , *p* = 0.001) (figure 5*a*). Dunn's post hoc test revealed that this main effect was owing to the time spent at looking the B-Cat being longer with respect to other stimuli: B-Cat (2.23 ± 0.13 (s); *m* ± s.e.m.) versus A-Cat-2 (0.55 ± 0.18 (s); *m* ± s.e.m.) and G-Background-1 (0.46 ± 0.13 (s); *m* ± s.e.m.) (*p* < 0.01); B-Cat versus G-Background-2 (0.31 ± 0.11 (s); *m* ± s.e.m.) and A-Cat-6 (0.66 ± 0.21 (s); *m* ± s.e.m.) (*p* < 0.05). No statistical significant differences were found between the other pairwise comparisons (*p* > 0.05, Dunn's post hoc test).

As to the behavioural score, the analysis revealed a main effect of the type of visual stimulus on the score for alerting behavioural response between visual stimuli ($\chi_{4}^{2}=18.648$, *p* < 0.01; Friedman's ANOVA, figure 5*b*). Dunn's post hoc test revealed that this main effect was owing to the score for alerting behavioural response to the B-Cat stimulus being higher with respect to other stimuli: B-Cat (3.75 ± 0.16 (s); *m* ± s.e.m.) versus G-Background-1 (0.44 ± 0.17 (s); *m* ± s.e.m.) (*p* < 0.01); B-Cat versus G-Background-2 (0.66 ± 0.11 (s); *m* ± s.e.m.), A-Cat-2 (0.62 ± 0.18 (s); *m* ± s.e.m.) and A-Cat-6 (0.75 ± 0.25 (s); *m* ± s.e.m.) (*p* < 0.05). No statistical significant differences were found between the other pairwise comparisons (*p* > 0.05, Dunn's post hoc test).

## 4. Discussion

Our results revealed that during presentations of a cat's moving animations having the same red–green colour shade of the number ‘2’ of Ishihara's plate no. 22 (i.e. RG-Cat-2), most of the dogs exhibit an orienting response to the stimulus (both the eyes and the head oriented toward the animated cat silhouette) together with clear targeting behaviour. These results are similar to those reported in red–green colour-blind humans who clearly recognize the red number ‘2’ during inspection of Ishihara's plate no. 22 and the cat during presentation of the RG-Cat-2 animation.

On the other hand, when dogs were presented with the red cat animation having the same red–green colour shade of the number ‘6’ of the Ishihara's plate no. 22, a significant lowering of both orienting and targeting behavioural response was observed, pointing to a considerable loss of subjects' capacities in perceiving the target.

The above result is in accordance with the difficulty of the red–green colour-blind humans to recognize the red-depicted number ‘6’ of Ishihara's plate no. 22 and their inability to perceive the cat movements during presentation of the RG-Cat-6 animation.

Furthermore, two aspects should be considered when the orienting response is used to evaluate visual discrimination: (i) habituation to the movements of the target (i.e. false negative response) and (ii) aspecific head/eyes orienting response (i.e. false positive response). Given that in the orienting response, habituation to the movements of the targets presented as the first could decrease the response to the next stimulus, we designed the experiment so that the two coloured targets were presented only once to each subject and the control stimulus (i.e. the black cat silhouette presented against a white back ground) was presented as the last during each session. In order to deal with the possibility of a ‘false positive response’, we scored during the experiment the targeting–alerting dogs behavioural response towards the coloured cat animations which was then compared with the behavioural response towards the control cat animation (B-Cat). A statistical significant correlation between the alerting–targeting behavioural response towards the coloured cat animation and the control ones make the hypothesis that dogs recognize the moving cat likely. In addition, in support to this hypothesis the behavioural score for alerting–targeting towards the control cat animation was higher compared with that of the black square, indicating that the subjects were able to clearly distinguish the movements of the cat from those of the square.

Regarding the dog–owner dyads, the possibility that the owners influenced the dogs' orienting response during the test is very remote because, although the dyad was very close during the test (the dog sat or was laid between the owner's legs), the owners were asked to stare a fixed spot centrally located to the screen. In addition, given the results, it is reasonable to assume, that the owners did not influence the dogs, because they otherwise might have reacted to the animations that must have been visible to the owners (i.e. both RG-Cat-2 and RG-Cat-6 stimuli because all the owners have normal colour vision).

Another aspect to consider is that the luminance difference between stimulus colours and background could affect colour discrimination thresholds [13]. Despite the fact that a modified version with the same colour characteristics of the Ishihara plate was used (already validated to test human deuteranopia) and that the brightness discrimination in dogs is about two times worse than in humans [7], the hypothesis that subjects have used achromatic cues to recognize the moving cat stimulus cannot be excluded. However, the results of the achromatic test clearly show that this is very unlikely, because when presented with the achromatic versions of the RG-Cat stimuli (i.e. A-Cat-2 and A-Cat-6) there were no differences in terms of both the looking time and the targeting behavioural response between cat stimuli and backgrounds. In other words, both the orienting and the targeting behavioural responses towards the cat's moving animations having the same red–green colour shade of the number ‘2’ of Ishihara's plate no. 22, decrease considerably when its achromatic version was presented to dogs.

This finding supported previous results demonstrating that, in dogs, colour information may be predominant with respect to brightness [9].

In addition, the observed difference between the red–green blindness test and the achromatic one is consistent with reports of compelling evidence that colour can contribute to motion perception [14–18], but is in contrast with recent other studies that have shown the lack of such a relationship [19]. Specifically, it has been suggested that at least two different mechanisms for processing motion exist: the first one that is extremely sensitive to colour (which was probably the one used in our experiment to perceive the RG-Cat-2 animation), which is engaged mainly with slow speeds, while the second one treats colour signals like low-contrast luminance signals and it is engaged with faster speeds [14,18]. That being said, it would be extremely interesting to check in future studies if the contribution of colour cues to the detection of the cat's motion is altered by the animation speed.

Overall, our results, together with the above-reported studies, confirm that dog colour vision is dichromatic in its nature, resembling that of human red–green blindness. Besides contributing to increasing knowledge about the perceptual ability of dogs, the present work showed, to our knowledge, for the first time, directly canine red–green blindness by using a modified test of colour vision in humans (Ishihara's test), thereby allowing direct comparison to colour vision (and colour blindness) in humans. Furthermore, the method used will open the door to the development of new techniques (e.g. coupling the modified version of the Ishihara's test with the use of eye-gaze detection and tracking systems) to assess colour vision in the animal kingdom.

## Data accessibility

The datasets supporting this article have been uploaded as the electronic supplementary material.

## References

[1] Mowat FM, Petersen-Jones SM, Williamson H, Williams DL, Luthert PJ, Ali RR, Bainbridge JW 2008 Topographical characterization of cone photoreceptors and the area centralis of the canine retina. Mol. Vis. 14, 2518–2527. 19112529 PMC2610288

[2] Mellersh CS et al. 2006 Canine RPGRIP1 mutation establishes cone-rod dystrophy in miniature longhaired dachshunds as a homologue of human Leber congenital amaurosis. Genomics 88, 293–301. (doi:10.1016/j.ygeno.2006.05.004) 16806805 10.1016/j.ygeno.2006.05.004

[3] Aguirre G 1978 Retinal degeneration in the dog: rod dysplasia. Exp. Eye Res. 26, 233–253. (doi:10.1016/0014-4835(78)90072-6) 639877 10.1016/0014-4835(78)90072-6

[4] Odom JV, Bromberg NM, Dawson WW 1983 Canine visual acuity: retinal and cortical field potentials evoked by pattern stimulation. Am. J. Physiol. I. 245, R637–R641. 10.1152/ajpregu.1983.245.5.R637 6638211

[5] Guyton AC, Hall JE 1999 Textbook of medical physiology, pp. 637–650. Philadelphia, PA: Elsevier Health Science.

[6] Pongrácz P, Ujvári V, Faragó T, Miklósi Á, Péter A 2017 Do you see what I see? The difference between dog and human visual perception may affect the outcome of experiments. Behav. Process. 140, 53–60. (doi:10.1016/j.beproc.2017.04.002) 10.1016/j.beproc.2017.04.002 28396145

[7] Pretterer G, Bubna-Littitz H, Windischbauer G, Gabler C, Griebel U 2004 Brightness discrimination in the dog. J. Vis. 6, 241–249. (doi:10.1167/4.3.10) 10.1167/4.3.10 15086313

[8] Rosengreen A 1969 Experiments in color discrimination in dogs. Acta Zool. Fenn. 121, 3–19.

[9] Kasparson AA, Badridze J, Maximov VV 2013 Colour cues proved to be more informative for dogs than brightness. Proc. R. Soc. B 280, 20131356 (doi:10.1098/rspb.2013.1356) 10.1098/rspb.2013.1356 PMC3730601 23864600

[10] Kelber A1, Vorobyev M, Osorio D 2003 Animal colour vision--behavioural tests and physiological concepts. Biol. Rev. 78, 81–118. (doi:10.1017/S1464793102005985) 12620062 10.1017/s1464793102005985

[11] Neitz J, Geist T, Jacobs GH 1989 Color vision in the dog. Vis Neurosci. 3, 119–125. (doi:10.1017/S0952523800004430) 2487095 10.1017/s0952523800004430

[12] Campbell TG, Lehn A, Blum S, Airey C, Brown H 2016 iPad colour vision apps for dyschromatopsia screening. J. Clin. Neurosci. 29, 92–94. (doi:10.1016/j.jocn.2015.10.042) 26896905 10.1016/j.jocn.2015.10.042

[13] Witzel C, Gegenfurtner KR 2013 Categorical sensitivity to color differences. J. Vis. 13, 1 (doi:10.1167/13.7.1) 10.1167/13.7.1 23732118

[14] Derrington AM 2000 Can colour contribute to motion? Curr. Biol. 10, R268–R270. (doi:10.1016/S0960-9822(00)00403-6) 10753737 10.1016/s0960-9822(00)00403-6

[15] Dougherty RF, Press WA, Wandell BA 1999 Perceived speed of colored stimuli. Neuron 24, 893–899. (doi:10.1016/S0896-6273(00)81036-3) 10624952 10.1016/s0896-6273(00)81036-3

[16] Wandell BA, Poirson AB, Newsome WT, Baseler HA, Boynton GM, Huk A, Gandhi S, Sharpe LT 1999 Color signals in human motionselective cortex. Neuron 24, 900–909. (doi:10.1016/S0896-6273(00)81037-5) 10.1016/s0896-6273(00)81037-5 10624953

[17] Seidemann E, Poirson AB, Wandell BA, Newsome WT 1999 Color signals in area MT of the macaque monkey. Neuron 24, 911–917. (doi:10.1016/S0896-6273(00)81038-7) 10624954 10.1016/s0896-6273(00)81038-7

[18] Gegenfurtner KR, Hawken MJ 1996 Interaction of motion and color in the visual pathways. Trends Neurosci. 19, 394–401. (doi:10.1016/S0166-2236(96)10036-9) 8873357 10.1016/S0166-2236(96)10036-9

[19] Yamaguchi S, Wolf R, Desplan C, Heisenberg M 2008 Motion vision is independent of color in *Drosophila*. Proc. Natl Acad. Sci. USA 105, 4910–4915. (doi:10.1073/pnas.0711484105) 18353989 10.1073/pnas.0711484105 PMC2290790

